# The Factorial Structure, Reliability, and Validity of a Coping Measure Among Women with HIV and Sexual Trauma in Cape Town, South Africa

**DOI:** 10.1007/s10461-025-04886-6

**Published:** 2025-09-23

**Authors:** Stephan Rabie, Anubhuti Poudyal, Amaleah Mirti, Patrick Wilson, John A. Joska, Kathleen J. Sikkema

**Affiliations:** 1 HIV Mental Health Research Unit, Neuroscience Institute, Department of Psychiatry and Mental Health, University of Cape Town, Anzio Road Observatory, Cape Town, 7925 South Africa; 2 Department of Sociomedical Sciences, Columbia Mailman School of Public Health, New York, NY USA; 3 Department of Psychology, University of California Los Angeles, Los Angeles, CA USA

**Keywords:** Coping, HIV, Trauma, Factor analysis, Psychometric properties, Women

## Abstract

The dual epidemics of HIV and sexual violence against women complicate HIV care engagement in South Africa. In settings with limited mental health treatment, robust measures to enhance screening and intervention for mental health specific to people with HIV are needed. The current study developed a 42-item assessment for coping with HIV and trauma in isiXhosa. In a randomized controlled trial, 350 participants completed measures of coping, anxiety, traumatic stress, and depression at their baseline assessments. Participants were women with HIV residing in Khayelitsha, South Africa, initiating or re-initiating antiretroviral therapy (ART) and histories of sexual abuse and trauma symptoms. We performed exploratory factor analysis with oblique rotation using parallel analysis and calculated Cronbach’s alpha for each coping factor to assess internal consistency. Finally, we evaluated convergent and discriminant validity among the subsequently identified factors and assessments of depression, traumatic stress, and anxiety. The parallel analysis suggested the retention of three factors: active, avoidant, and social/spiritual coping. The final measure included 39 items with an overall Cronbach’s Alpha of 0.825. The factor structure demonstrated robust goodness of fit. Convergent validity was demonstrated through significant positive correlations between active and social/spiritual coping, and between avoidant coping and the depression, anxiety, and traumatic stress. Significant negative correlations of active and social/spiritual coping with depression, anxiety, and traumatic stress scores also supported the discriminant validity between subscales.We conclude that the Coping with HIV measure provides a strong and validated measure of coping for use in populations in South Africa living with HIV.

*Clinical Trial Number*: NCT04793217.

**Supplementary Information:**

The online version contains supplementary material available at 10.1007/s10461-025-04886-6.

## Introduction

Among the 7.7 million people currently living with HIV in South Africa, 4.9 million are women [1]. Women account for over 60% of new HIV infections, and those aged 15 and older have an HIV prevalence rate of 22.6 compared to 17.1 among men in the same age group [1]. These disproportionately high rates of HIV acquisition and prevalence among women stem from a range of structural, behavioral, and biological factors [2]. Factors contributing to increased HIV acquisition and prevalence among women, such as structural inequities, poverty, and traumatic experiences, such as intimate partner violence [2, 3] and sexual trauma [4, 5], also impact HIV-related outcomes, including engagement in care and viral suppression [6]. Similar stressors are linked to poor mental health outcomes, including higher rates of depression, anxiety, and traumatic stress [7], which further aggravate outcomes for individuals across the HIV-care continuum, from preventive behaviors and HIV testing to healthcare engagement, disease progression, antiretroviral treatment (ART) adherence, and viral suppression [8–11].

People with HIV often experience stigma and associated feelings of anger, denial, and shame related to their HIV diagnosis [12–14] and employ various strategies to cope with the psychological burden of these feelings. In this context, the coping strategies employed to deal with the intersecting physical and psychological stressors are crucial to improving HIV-related outcomes among women with HIV. Coping strategies are the cognitive and behavioral efforts used to deal with stressful and often resource-demanding events [12–14]. There is evidence of gender differences in the magnitude of effect for both environmental and symptom-based stressors [15, 16], which makes it essential to understand coping strategies among women with HIV. Multiple studies have reported the influence of adaptive and maladaptive coping strategies on HIV and mental health outcomes [15, 17, 18]. For example, adaptive coping strategies, such as active coping and spiritual/religious coping, are associated with improved ART adherence [17]. Similarly, among women with HIV, those who employ avoidant or disengaged coping, and report social isolation have higher anxiety and depression symptoms [15, 19]. Avoidant coping has also been associated with lower CD4 count and viral load changes [20, 21].

Coping scales such as the Brief COPE [22], Ways of Coping (WOC) [23], and Coping with Illness (CWI) [24] tools have been extensively utilized to assess coping strategies among people with HIV [25–27]. However, the development of measures to understand coping mechanisms among women with HIV in South Africa should be guided by contextual knowledge of coping strategies and the identification of relevant items from validated tools. Thus, for our prior work in South Africa [28, 29], we adapted items from the abovementioned validated scales and added new items derived from cognitive interviewing to develop a comprehensive coping scale. In the current paper, we administered this tool to women with HIV who have experienced sexual trauma in Cape Town, South Africa. Here, we report the psychometric properties of this coping scale, including its factorial structure and internal consistency. We also examine convergent and discriminant validity through Pearson correlations between the general factors of the coping measure and the Center for Epidemiological Studies Scale (CES-D), anxiety subscale of the Brief Symptoms Inventory (BSI), and the Posttraumatic Stress Disorder Checklist for DSM-5 (PCL-5).

## Methods

### Study Setting and Sample

This study uses baseline survey data from *Project Someleze*, a hybrid type 1 effectiveness-implementation randomized controlled trial evaluating the effectiveness of a coping intervention among women with histories of HIV and sexual trauma [30]. The study was conducted in Khayelitsha, a peri-urban settlement on the outskirts of Cape Town, South Africa. Khayelitsha carries the largest HIV burden in the region and is characterized by pervasive unemployment, poor infrastructure, and high rates of violence. Data were collected from four primary healthcare facilities in Khayelitsha. Each facility offers comprehensive HIV care and serves between 7500 and 10,000 patients with HIV – two-thirds of these patients are female. Universal test and treat guidelines were introduced in South Africa in 2016, and ART initiation at the clinic follows the standard protocol per government guidelines.

All women who received a positive HIV diagnosis or presented themselves for ART initiation or re-initiation (i.e., returning to care 30 days or more after interruption in treatment) were identified by clinic staff and referred to study staff for eligibility screening. In a private office, English and isiXhosa-speaking study staff administered oral consent for the initial screening, emphasizing confidentiality and the sensitive nature of the screening items. All participants were able to understand the assessments. The brief assessment included screening items on demographic characteristics, depression, intimate partner violence, sexual trauma, traumatic stress symptoms, and suicide risk. Participants were eligible for enrollment if they met the following criteria: (1) Living with HIV and between 2 weeks and 4 months since initiation of first-line ART (as new initiators or re-initiators); (2) history of sexual abuse or assault during childhood, adolescence, or adulthood; (3) endorsement of any symptoms of post-traumatic stress; (4) 18 years or older; (5) English and/or isiXhosa-speaking; and (6) receiving HIV care services at a healthcare facility. Patients were excluded and referred to psychiatric services if they met the criteria for high risk of suicide. Additional exclusion criteria included patients initiated on second- or third-line ART, the inability to provide informed consent, or the inability to communicate in isiXhosa or English.

### Data Collection

Baseline data were collected between February 2021 and April 2024. Baseline assessments were completed between two weeks and four months since ART initiation (or re-initiation). The baseline assessments were administered by experienced research assistants and supervised by a PhD-level research psychologist. The baseline assessment was conducted in private offices located at the healthcare facilities and administered by means of an individual interview in isiXhosa or English, based on participant preference. Participants were given opportunities to ask questions and clarifications during the baseline assessment. Each assessment was completed between 45 and 60 min, using Research Electronic Data Capture (REDCap; UL1TR001873) and mobile tablets.

### Measures

The measures from the full assessment battery used in the current study include a demographic survey, the Coping with HIV and Trauma measure, the Anxiety dimension of the Brief Symptom Inventory (BSI) [31], the Center for Epidemiology Studies – Depression (CES-D) [32], and the Posttraumatic Stress Disorder Checklist for DSM-5 (PCL-5) [33].

#### Demographic Characteristics

We collected information on participants’ age, race, pregnancy status, level of education, employment status, ART initiation status, and ART regimen.

#### Sexual Abuse and Intimate Partner Violence

The inclusion criteria of lifetime history of sexual abuse and recent sexual abuse (defined as sexual abuse or assault that occurred within the past 4 months), was assessed at screening using three items based on the WHO Composite International Diagnostic Interview (CIDI) [34]. Example items include “Did anyone touch you in a sexual way or make you touch them in a sexual way against your will, when you made it clear through words or actions that you did not want to, or when you were afraid to say no?”. Responses were dichotomized into “yes/no” (yes = positive endorsement any of the items) to reflect experiences of sexual abuse. Physical intimate partner violence (IPV) occurring in adolescence or adulthood, and within the past 4 months, was measured using five items from the South African adaptation of the physical IPV subscale of the revised Conflict Tactics Scale (CTS2) [35]. Example items include “Has a partner ever beat, kicked or hit you?”. Any endorsement of these items reflected experiences of physical IPV during either adolescence or adulthood, with recent physical IPV defined as endorsing experience of items in the past 4 months.

#### Coping

Coping was assessed using 42 items, previously used in our pilot study [28]. The measure was developed using items previously used to measure coping in similar populations in Sub-Saharan Africa [22–24, 22–24]. Five items (2, 3, 19, 25, and 26) were adapted from the Brief Cope measure [22]; 19 items (4, 6, 8, 9, 10, 11, 18, 21, 23, 24, 27, 29, 30, 34, 40, 41, 43, 46, 47) from the Ways of Coping Questionnaire [23]; and six items (5, 20, 32, 33, 37, 42) from the Coping with Illness measure [24]. Four items (12, 13, 38, 39) overlapped across measures. Finally, eight items (1, 14, 16, 17, 35, 36, 44, 45) were added in the pilot study based on cognitive interviewing and formative findings. Our prior study [29] measured coping using similar items in a sample of 64 women with HIV and sexual trauma histories attending primary care in Cape Town, South Africa. Our pilot study demonstrated sufficient reliability (Cronbach’s alpha = 0.78) [36].

We used a forward-back translation approach to develop the items in isiXhosa [37]. Two bilingual, isiXhosa-speaking research assistants with backgrounds in psychology independently translated the measure. All items were developed using a constrained emic approach [38]. A third bilingual, isiXhosa-speaking translator reviewed the independent translations and compared each item for accuracy. Inconsistencies were resolved through a panel meeting with the three translators, convened by the trial project director. Response options on the final items involved asking participants how often they used these strategies in the past month to help deal with their HIV illness (1 = not at all to 4 = most of the time).

#### Brief Symptom Inventory (BSI)

A subscale of the Brief Symptom Inventory was used to assess anxiety. The Anxiety subscale on the BSI comprises six items, and participants are asked how much they have been bothered by symptoms of anxiety over the past month (0 = Not at all to 4 = Extremely). The BSI has been used in South African populations, including PWH, and the overall BSI has demonstrated good internal consistency (Cronbach’s alpha = 0.81) [39].

#### Center for Epidemiology Studies Depression (CES-D)

The Center for Epidemiology Studies – Depression was used to measure depression. The CES-D is a 20-item measure that asks respondents to rate how often they experienced symptoms of depression over the past week, such as poor appetite, restless sleep, and feeling lonely. Response options range from 0 (rarely or none of the time) to 3 (most or almost all the time) for each item. The CES-D has been used extensively in South African populations with demonstrated reliability (Cronbach’s alpha = 0.95) [37] and validity [40].

#### Posttraumatic Stress Disorder Checklist for DSM-5 (PCL-5)

The Validated Posttraumatic Stress Disorder Checklist for DSM-5 [33] was used to assess the severity of traumatic stress. The measure includes 20 items with response options ranging from 1 (Not at all) to 5 (Extremely). Possible scores ranged from 20 to 100, with higher scores indicating higher traumatic stress levels. Prior studies in South Africa have reported Cronbach’s alpha of 0.95 for PCL-5 [41].

### Data Analysis

Frequency statistics were computed to describe the sample and participants’ history of sexual abuse and physical IPV. The following analytical procedures were employed to establish the factorial structure described by the coping items. Since factors were expected to be correlated, an exploratory factor analysis (EFA) with oblique rotation (Promin) was conducted on the items. We utilized parallel analysis, which compared the observed eigenvalues produced by the EFA to random datasets to estimate the number of factors to retain. The EFA yielded a pattern matrix, which was analyzed to identify items with factor loadings below |0.30| or items with cross-loadings exceeding |0.30|. These items were flagged for subsequent analyses to establish the underlying factorial structure of the coping items. Subsequently, an EFA with a semi-specified orthogonal Procrustes rotation (i.e., target rotation), using 95% bootstrapped confidence intervals, was applied to the items. This target rotation maximizes factor loadings to illustrate the item correlation with the latent factor. Since the coping items are scored on a four-point Likert scale, this EFA employed Robust Unweighted Least Squares (RULS) as the factor estimator. Finally, a LOSEFER empirical correction was applied to the items to generate Robust Goodness of Fit statistics, including the Root Mean Square Error of Approximation (RMSEA; < 0.08) [42]; Non-Normed Fit Index (NNFI; >0.90) [43]; Comparative Fit Index (CFI; >0.90) [44]; and the LOSEFER empirically corrected Chi-square *(p <* 0.05) [45]. We also evaluated internal consistency using Cronbach’s alpha, with values ≥ 0.70 considered acceptable, ≥ 0.80 as good, and ≥ 0.90 as excellent [36]. We also calculated McDonald’s omega, with values > 0.70, indicating acceptable reliability (supplementary table) [46]. To examine convergent and discriminant validity, we computed Pearson correlations among the three coping scales, BSI, CES-D, and PCL-5. Convergent validity was analyzed by assessing correlations between theoretically related constructs, while discriminant validity was examined through correlations between conceptually distinct constructs. We used FACTOR Version 12.04.05 for the factor analysis and *R* Version 4.4.2 for the reliability and validity analyses.

## Results

We screened 1400 women for trial eligibility, of which 510 participants met the inclusion criteria for the trial. In total, 350 eligible participants completed baseline assessments. Five participants withdrew during the course of the trial, resulting in a final *n* = 345 used in the current analysis. The mean age of the sample was 33.1 years (SD = 9.3), ranging between 18 and 64 years of age. The majority of participants (98.9%; *n* = 341) identified their race as Black African. 18% (*n* = 63) of participants were pregnant at the time of assessment. Nearly two-thirds (74.6%; *n* = 261) of participants were unemployed at baseline, with an average monthly income of ZAR1158 (approximately $65 USD). In terms of initiation status, most participants were re-initiators (64.3%); with 35.7% new initiators. As part of inclusion criteria, all participants (100%; *n =* 350) had a lifetime history of sexual abuse. In addition, 77% (*n =* 264) of participants experienced sexual abuse during adulthood (26.9% of participants experienced sexual abuse during both childhood and/or adolescence and adulthood). In addition, 18% (*n =* 66) reporting recent sexual abuse (i.e., in the past 4 months). 23% of participants reported sexual abuse during childhood or adolescence only. Although not an inclusion criterion, 75% (*n* = 260) of participants reported experiences of physical IPV during adulthood, with 44% (*n =* 64) of participants reporting recent physical IPV (i.e., in the past 4 months).

Four participants had missing data for one of the coping items and were excluded from the analysis. Based on 42 items and 341 participants across 100 random simulations, the parallel analysis suggested retaining three factors. After computing the EFA with Promin rotation, the pattern matrix was examined. Three items failed to load on any factor based on our critical factor of 0.30 (COP16 = 0.223; COP30 = 0.286; COP32 = 0.255), although the loadings were approaching the 0.30 cut-off coefficient. There were no item cross-loading greater than 0.30, however, one item (COP32) cross-loaded on two factors (F2 = 0.280; F3 = 0.255). These items were flagged for further inspection in the target rotation EFA. At this point, a clear, simple structure emerged from the data, and based on the items that loaded together, the factors were labeled as Active Coping, Avoidant Coping, and Social/Spiritual Coping.

The target rotation EFA was performed to confirm the item loadings on the three identified factors. Importantly, the item loadings of COP 16, COP30 and COP32 (flagged in Promin rotated EFA) did not improve in the target rotation EFA to meet the critical factor threshold of 0.30. Similarly, COP32 cross-loaded on two factors (F2 = 0.272; F3 = 0.261), although these cross-loadings were not greater than 0.30. These results indicated that the three items did not correlate with the respective latent factors and were subsequently removed. The factor analysis was rerun with the remaining 39 items, and no further changes were made. At this point, all items loaded uniquely onto a single factor.

The target rotation EFA also produced an inter-factor correlation matrix for the three factors. As conceptually expected, a moderate positive correlation was observed between Factor 1: Active Coping and Factor 3: Social/Spiritual Coping (*r* = 0.533). In addition, a weak negative correlation was observed between Factor 1: Active Coping and Factor 2: Avoidant Coping (*r* = − 0.024), and a weak positive correlation between Factor 2: Avoidant Coping and Factor 3: Social/Spiritual Coping (*r* = 0.170). These results are presented in Table 1.

**Table 1:** Inter-factor correlation matrix

	Factor 1: Active Coping	Factor 2: Avoidant Coping	Factor 3: Social/Spiritual Coping
Factor 1: Active Coping	1.00		
Factor 2: Avoidant Coping	− 0.024	1.00	
Factor 3: Social/Spiritual Coping	0.533	0.170	1.00

Table 2 shows the final factor loading for the target rotation EFA three-factor solution. The target rotation EFA also produced evidence for the Goodness of Fit of the factor structure. Specifically, the RMSEA = 0.066 (*p* = 1.00) demonstrated acceptable model fit, while the NNFI = 0.919 and CFI = 0.930 met the threshold of 0.90 to demonstrate goodness of fit. The LOSEFER empirically corrected Chi-square (c2 = 918.42; *p* = 0.0001) demonstrated robust goodness of fit.

**Table 2:** Final factor loading for the target rotation EFA three-factor solution*

Scale label	Scale item	Factor 1: Active coping	Factor 2: Avoidant coping	Factor 3: Social/spiritual coping
COP1	I forgave myself for mistakes I’ve made.	**0.473**	−0.026	0.158
COP2	I looked at the situation from another side, to see if it could lead to something positive.	**0.572**	0.002	0.102
COP3	I have been accepting the reality of the fact that it has happened, and I am learning to live with it.	**0.675**	−0.207	0.035
COP4	I told myself positive words that helped me feel better.	**0.784**	−0.154	−0.119
COP5	I thought a lot about what is really important in my life.	**0.733**	−0.124	−0.096
COP6	I changed something so things would turn out alright.	**0.668**	0.144	−0.06
COP8	I knew what had to be done, so I worked even harder to make things work.	**0.851**	−0.023	−0.068
COP9	I came up with a couple of different solutions to the problem.	**0.695**	0.047	0.102
COP10	I broke the problem down into smaller pieces in order to understand it better.	**0.737**	0.075	0.048
COP11	I changed something about myself.	**0.613**	0.152	0.039
COP12	I have been thinking hard about what steps to take.	**0.621**	0.077	−0.032
COP13	I made a plan and have taken steps to improve the situation.	**0.716**	0.115	0.053
COP17	I focused on being there for my family.	**0.325**	−0.103	0.088
COP18	I criticized myself.	−0.092	**0.695**	0.081
COP19	I’ve been blaming myself for things that happened.	0.011	**0.724**	−0.005
COP20	I’ve been thinking too much about the problem and couldn’t stop thinking about it.	−0.079	**0.696**	0.023
COP21	I bottled my feelings inside.	−0.04	**0.712**	−0.118
COP23	I felt resigned, since I thought that nothing could be done.	0.013	**0.468**	0.054
COP24	I tried to push the thing from my mind.	0.085	**0.581**	−0.026
COP25	I’ve been giving up trying to deal with it.	−0.019	**0.687**	0.083
COP26	I’ve been saying to myself “this isn’t real.”	0.03	**0.67**	−0.11
COP27	I’ve wished that the situation would go away or somehow be over with.	−0.019	**0.679**	−0.052
COP29	I’ve wished that I could change what happened or how I felt.	0.007	**0.673**	−0.073
COP42	I planned ways to hurt myself.	−0.011	**0.472**	0.141
COP43	I tried to make myself feel better by drinking or using drugs.	0.005	**0.341**	−0.044
COP44	I did things to avoid addressing my problems (e.g. watched TV, read, or slept).	0.298	**0.408**	0.013
COP45	I used sex to keep from feeling bad or to get what I needed.	−0.026	**0.325**	0.111
COP46	I treated someone badly who did not deserve it because I was feeling bad.	−0.004	**0.356**	0.164
COP47	I generally avoided being with people.	−0.101	**0.487**	−0.156
COP14	I found opportunities to help or encourage other women like myself.	0.062	0.009	**0.596**
COP33	I went to a place of worship or participated in church activities (e.g., women’s groups, choir).	−0.095	0.03	**0.457**
COP34	I prayed to find strength or guidance	0.184	0.085	**0.340**
COP35	I read religious writings or listened to religious teachings, for example, a spiritual radio program, for inspiration.	0.134	0.127	**0.384**
COP36	I talked to a member of the clergy (priest, minister, Imam, etc.)	−0.032	0.052	**0.551**
COP37	I talked with others with problems like mine.	−0.027	−0.065	**0.621**
COP38	I talked to a therapist, counselor, social worker or support group about my problems.	−0.074	0.118	**0.574**
COP39	I’ve been getting sympathy or understanding from someone.	−0.127	−0.08	**0.657**
COP40	I asked a relative, elder, or friend I respected for advice.	−0.073	−0.093	**0.791**
COP41	I talked to someone to find out more about the situation.	−0.047	0.000	**0.756**

Original sources of each of the COP items: Brief Cope – COP items (2, 3, 12**, 13**, 19, 25, 26, 39**) Ways of coping [23] – COP items (4, 6, 8, 9, 10, 11, 12**, 13**, 18, 21, 23, 24, 27, 29, 30, 34, 38**, 39**, 40, 41, 43, 46, 47) Coping with Illness [24]– COP items (5, 20, 32, 33, 37, 38**, 42)

New/adapted items (added in pilot study) – COP items (1, 14, 16, 17, 35, 36, 44, 45)

*Bolded items indicate strongest loading on each factor

**Overlap of items across measure

### Internal Consistency

The standard Cronbach’s alpha coefficient for active, avoidant, and social/spiritual coping subscales were 0.907, 0.883, and 0.820, respectively, indicating good to excellent internal consistency values (Table 3). Additionally, the McDonald’s omega total for the three scales were 0.91, 0.89, and 0.83, indicating good to excellent reliability for the three subscales.

**Table 3:** Alpha coefficients (Cronbach) for internal consistency of the coping subscales

		Scale Mean if Item Deleted	Corrected Item-Total Correlation	Cronbach’s Alpha if Item	Mean ± SD
*Active coping*					
COP1	I forgave myself for mistakes I’ve made.	39.796	0.541	0.904	3.34 ± 1.04
COP2	I looked at the situation from another side, to see if it could lead to something positive.	39.799	0.609	0.901	3.34 ± 1.02
COP3	I have been accepting the reality of the fact that it has happened and I am learning to live with it.	39.568	0.655	0.899	3.57 ± 0.82
COP4	I told myself positive words that helped me feel better.	39.452	0.681	0.898	3.69 ± 0.68
COP5	I thought a lot about what is really important in my life.	39.456	0.648	0.899	3.68 ± 0.69
COP6	I changed something so things would turn out alright.	39.920	0.580	0.902	3.22 ± 1.14
COP8	I knew what had to be done, so I worked even harder to make things work.	39.692	0.762	0.894	3.45 ± 0.95
COP9	I came up with a couple of different solutions to the problem.	40.169	0.711	0.896	2.97 ± 1.2
COP10	I broke the problem down into smaller pieces in order to understand it better.	40.169	0.728	0.896	2.97 ± 1.23
COP11	I changed something about myself.	40.114	0.601	0.901	3.03 ± 1.25
COP12	I have been thinking hard about what steps to take.	39.764	0.577	0.902	3.38 ± 1.05
COP13	I made a plan and have taken steps to improve the situation.	39.995	0.689	0.897	3.14 ± 1.15
COP30	I went on as if nothing had happened.	39.785	0.316	0.913	3.36 ± 1.06
Overall	**Cronbach Alpha (Active coping)**	**0.907**			
*Avoidant coping*					
COP18	I criticized myself.	41.144	0.661	0.870	2.88 ± 1.31
COP19	I’ve been blaming myself for things that happened.	40.875	0.671	0.870	3.14 ± 1.15
COP20	I’ve been thinking too much about the problem and couldn’t stop thinking about it.	41.000	0.656	0.871	3.02 ± 1.23
COP21	I bottled my feelings inside.	41.034	0.649	0.871	2.99 ± 1.32
COP23	I felt resigned, since I thought that nothing could be done.	40.705	0.449	0.879	3.32 ± 1.09
COP24	I tried to push the thing from my mind.	40.663	0.540	0.875	3.36 ± 1.05
COP25	I’ve been giving up trying to deal with it.	41.378	0.643	0.871	2.64 ± 1.32
COP26	I’ve been saying to myself “this isn’t real.”	41.086	0.604	0.873	2.93 ± 1.32
COP27	I’ve wished that the situation would go away or somehow be over with.	40.626	0.622	0.872	3.39 ± 1.09
COP29	I’ve wished that I could change what happened or how I felt.	40.546	0.604	0.873	3.47 ± 1.01
COP42	I planned ways to hurt myself.	42.219	0.487	0.878	1.8 ± 1.19
COP43	I tried to make myself feel better by drinking or using drugs.	42.037	0.348	0.883	1.98 ± 1.28
COP44	I did things to avoid addressing my problems (e.g. watched TV, read, or slept).	40.869	0.365	0.883	3.15 ± 1.11
COP45	I used sex to keep from feeling bad or to get what I needed.	42.526	0.349	0.883	1.49 ± 1.04
COP46	I treated someone badly who did not deserve it because I was feeling bad.	42.029	0.398	0.881	1.99 ± 1.29
COP47	I generally avoided being with people.	41.558	0.451	0.879	2.46 ± 1.31
Overall	**Cronbach Alpha (Avoidant coping)**	**0.883**			
*Social/spiritual coping*					
COP14	I found opportunities to help or encourage other women like myself.	30.240	0.551	0.806	1.94 ± 1.23
COP16	I found motivation in my children or children around me.	29.340	0.291	0.826	2.84 ± 1.3
COP17	I focused on being there for my family.	28.680	0.274	0.827	3.5 ± 0.9
COP32	I trusted my belief in God.	28.550	0.416	0.817	3.63 ± 0.73
COP33	I went to a place of worship or participated in church activities (e.g., women’s groups, choir).	29.940	0.426	0.816	2.24 ± 1.32
COP34	I prayed to find strength or guidance	28.790	0.494	0.811	3.39 ± 0.96
COP35	I read religious writings or listened to religious teachings, for example, a spiritual radio program, for inspiration.	29.190	0.474	0.812	2.99 ± 1.26
COP36	I talked to a member of the clergy (priest, minister, Imam, etc.)	30.630	0.495	0.811	1.55 ± 1.09
COP37	I talked with others with problems like mine.	30.400	0.503	0.810	1.78 ± 1.17
COP38	I talked to a therapist, counselor, social worker or support group about my problems.	30.450	0.459	0.813	1.73 ± 1.22
COP39	I’ve been getting sympathy or understanding from someone.	29.910	0.474	0.812	2.27 ± 1.28
COP40	I asked a relative, elder, or friend I respected for advice.	30.010	0.608	0.802	2.17 ± 1.32
COP41	I talked to someone to find out more about the situation.	30.020	0.595	0.803	2.16 ± 1.33
Overall	Cronbach Alpha (Social/Spiritual coping)	**0.820**			

### Convergent and Discriminant Validity

Table 4 provides a descriptive summary of the three coping scales (active, avoidant, and social/spiritual) and measures of psychological distress - anxiety (BSI) and depression (CES-D). We conducted Pearson correlation analyses (Table 5) to assess convergent and discriminant validity among the three coping subscales, BSI, CES-D, and PCL-5.

**Table 4:** Overall summary of the coping subscales, BSI, and CES-D scales

	Mean ± SD	Median	IQR	Min	Max
Active Coping subscale	43.2 ± 9.1	46.0	38.0–51.0	13.0	52.0
Avoidant Coping subscale	43.9 ± 11.5	46.0	37.0–52.0	16.0	64.0
Social/Spiritual Coping subscale	32.2 ± 8.7	31.0	26.0–37.0	16.0	52.0
BSI	7.3 ± 6.5	6.0	1.0–12.0	0.0	24.0
CES-D	25.9 ± 14.5	27.0	15.0–36.0	0.0	58.0
PCL-5	39.7 ± 19.1	40.0	25.0–54.0	0.0	80.0

**Table 5:** Pearson correlations between coping subscales, BSI, and CES-D scales

	Active coping	Avoidant coping	Social/Spiritual Coping	BSI	CESD	PCL
1. Active coping	1.000	-	-	-	-	
2. Avoidant coping	0.018	1.000	-	-	-	
3. Social/Spiritual coping	0.514*	0.128*	1.000	-	-	
4. BSI	0.022	0.504*	0.204*	1.000	-	
5. CES-D	−0.108*	0.528*	0.032	0.709*	1.000	
6. PCL	0.038	0.502*	0.130*	0.591*	0.561*	1.000

*p < 0.05

#### Convergent Validity

The avoidant coping subscale demonstrated moderate positive correlations with the BSI (*r* = 0.504), CES-D (*r* = 0.528), and PCL-5 (*r* = 0.502), indicating good convergent validity. This aligns with theoretical expectations that avoidant coping strategies are associated with higher levels of psychological distress, depressive symptoms, and traumatic stress severity. Moreover, a strong positive correlation between the BSI and CES-D (*r* = 0.709), and moderately positive correlations between the BSI and PCL-5 (*r* = 0.591) and between the CES-D and PCL-5 (*r* = 0.561) reinforced the convergent validity of the measures of psychological distress in our sample. Lastly, the social/spiritual coping subscale was positively correlated with active coping (*r* = 0.514).

#### Discriminant Validity

Both the active coping and social/spiritual subscales exhibited weak correlations or no significant relationship with the BSI, CES-D, and PCL-5, indicating good discriminant validity. The weak correlation between the active and avoidant coping subscales (*r* = 0.018) and between social/spiritual and avoidant coping (*r* = 0.128) supported discriminant validity between these theoretically distinct coping strategies.

## Discussion

In this study, we examined the factorial structure, internal consistency, and convergent and discriminant validity of a coping measure administered to women with HIV who have experienced sexual trauma in Cape Town, South Africa. After removing three items from the original measure, a factor analysis was re-run with the remaining 39 items and revealed that all items loaded uniquely onto a single factor, leading to the identification of three coping subscales: active coping, avoidant coping, and social/spiritual coping. These subscales demonstrated good to excellent internal consistency and good convergent and discriminant validity, as assessed through the correlations between the three subscales and related measures, such as the BSI and CES-D.

Adherence to ART may be particularly challenging for women with trauma, as experiences of sexual trauma and related symptoms, such as dissociation and avoidance [15, 47, 48], may negatively impact HIV care engagement. When left unaddressed, avoidant coping can result in poor health-related behaviors, including retention in HIV care, engagement in risky sexual behavior, and non-adherence to treatment [15, 17, 18]. In this context, a reliable and valid measure of coping in the context of HIV and trauma is important. Our findings indicate that women with HIV and histories of sexual trauma utilize active, avoidant, and social/spiritual coping strategies. Existing research, including our earlier studies, suggests that active coping is linked to higher levels of HIV care engagement and adherence. For example, our pilot randomized controlled trial demonstrated that a culturally adapted brief coping intervention reduced traumatic symptoms and avoidant coping, while enhancing social and spiritual coping [29]. More recently, similar results have been reported in a US sample of older adults living with HIV, with behavioral disengagement (i.e., avoidant coping) found to be a significant predictor of poor ART adherence [49]. In South Africa, research suggests that avoidant coping is associated with a lower likelihood of ART initiation and viral suppression among people with HIV [20]. Our results demonstrate that a reliable and valid coping measure, such as the one reported in this study, can be used to identify individuals who employ maladaptive coping strategies when initiating ART. This represents an opportunity to intervene with individuals who struggle to cope with their HIV, and specifically to support women with HIV with histories of sexual trauma to adhere to ART and actively engage in HIV care.

In recent years, there have been growing calls for the inclusion of mediation analyses in mental health research to understand how interventions exert their influence and improve outcomes in patients [50]. However, the variables included in mediation analysis should be informed by theory and/or empirical evidence. In the context of previous research reporting the mediating role of improving coping to reduce traumatic stress and potentially enhance care engagement, it is crucial to assess coping by means of a reliable and valid measure with demonstrated psychometric properties. The Coping with HIV measure reported in this study provides support for its use in future mediation analyses, and importantly, to understand the underlying mechanisms of effective interventions. Importantly, while the measure reported in this study was developed specific to HIV, it holds potential for future adaptation and use in other populations and health conditions.

The associations reported in this study for the coping factors and psychological variables align with existing literature on coping with HIV. Crucially, these associations support the convergent and discriminant validity of the coping factors reported in this study. For example, the avoidant coping factor was positively correlated with both the anxiety and depression measures in this study, suggesting an increase in avoidant coping is associated with increased symptoms of anxiety and depression. The current findings indicate that participants who were experiencing depression or anxiety were significantly more likely to utilize avoidant coping strategies, while participants experiencing anxiety were more likely to utilize social/spiritual coping strategies. These results did not elucidate the directionality of this association. That is, it was unclear whether social coping was used in response to symptoms of anxiety or whether anxiety was experienced in response to social coping strategies. Similarly, although not significant, we found a negative correlation between active coping and depression, suggesting that individuals with depression symptoms were less likely to utilize active coping strategies.

This study has several limitations. Firstly, the cross-sectional design and selection of a female-only sample, limits the broader application of the underlying factorial structure reported in this study. Secondly, all data were obtained using self-report assessment, subject to response bias and the Hawthorne effect. Thirdly, this analysis did not assess item-level functioning, which could extrapolate items that perform poorly under each factor. This should be investigated in future studies. Finally, participants were recruited from primary healthcare facilities in Khayelitsha, Cape Town, and represent an already help-seeking population. Individuals with higher levels of avoidant coping are less likely to present for care at these facilities. This limitation limits the generalizability of the results. Despite these limitations, our study has expanded the scope of the coping with HIV literature by looking at coping strategies used by women with HIV with histories of sexual trauma. Considering the significant intersection of trauma and mental health concerns among women with HIV, this study contributes to our understanding of coping in this population. It highlights potential areas for intervention to improve adherence to ART and engagement in HIV care. Importantly, the development of a psychometrically sound coping measure provides the opportunity to determine the role of coping as a causal mechanism in mental health intervention trials, including those that examine clinical outcome such as viral load suppression.

## Conclusions

The Coping with HIV measure reported in the study demonstrate sound psychometric properties and an underlying factorial structure of three coping strategies: active, avoidant, and social/spiritual. The development of reliable and valid coping measure not only enhances our ability to screen for mental health concern among people with HIV but provides opportunities to determine the role of coping as a causal mechanism in mental health intervention trials, including those that examine clinical outcome such as viral load suppression. Finally, while this measure was developed for HIV-specific populations, it is suitable for adaptation and application in other populations and health conditions.

## Supplementary Information

Below is the link to the electronic supplementary material.

Supplementary material 1 (DOCX 14.1 kb)

## Data Availability

The data that support the findings of this study are available from the corresponding author upon reasonable request.
