# Supplementary material for: The Factorial Structure, Reliability, and Validity of a Coping Measure Among Women with HIV and Sexual Trauma in Cape Town, South Africa
Source: AIDS Behav. 2025 Sep 23;30(2):392–402. doi: 10.1007/s10461-025-04886-6 (PMC12929221; doi:10.1007/s10461-025-04886-6)
Supplement: Supplementary file 1 — Supplementary material 1 (DOCX 14.1 kb) [file 10461_2025_4886_MOESM1_ESM.docx]

**Histogram for fitted residuals**

Value Freq

|

-0.1673 2 |

-0.1205 24 | ***

-0.0736 92 | ***********

-0.0268 315 | ****************************************

0.0200 290 | ************************************

0.0668 98 | ************

0.1136 17 | **

0.1604 12 | *

0.2072 6 |

0.2541 2 |

0.3009 3 |

+-----------+---------+---------+-----------+

0 78.8 157.5 236.2 315.0

**Stemleaf Plot for Standardized Residuals**

-3 | 1

-2 | 74332222221110000

-1 | 99998888888777666655555555554444433333333333322222222222111111111111111111111100000000000000000000

-0 | 99999999999999999999988888888888888888888888888888777777777777777777777777777777777766666666666666666666666665555555555555555555555555555555544444444444444444444444444444444444444333333333333333333333333333333333333333333333333333333333322222222222222222222222222222222222222222211111111111111111111111111111111111111111

0 | 00000000000000000000000000000000000000000000000000000011111111111111111111111111111111111111222222222222222222222222222222223333333333333333333333333333333333344444444444444444444444444444444444555555555555555555555555555555555556666666666666666666666666666777777777777777777778888888888888888888888889999999999999999999999999

1 | 00000000001111111111222222222233333333344444445555555566666778999

2 | 00012223334667

3 | 012223333567899

4 | 78

5 | 356
